# Editorial: Exploring the Nature, Content, and Frequency of Intrapersonal Communication

**DOI:** 10.3389/fpsyg.2020.601754

**Published:** 2020-10-22

**Authors:** Thomas M. Brinthaupt, Alain Morin, Małgorzata M. Puchalska-Wasyl

**Affiliations:** ^1^Department of Psychology, Middle Tennessee State University, Murfreesboro, TN, United States; ^2^Department of Psychology, Mount Royal University, Calgary, AB, Canada; ^3^Institute of Psychology, Department of Personality Psychology, The John Paul II Catholic University of Lublin, Lublin, Poland

**Keywords:** self-talk, inner speech, intrapersonal communication, internal dialogue, imaginary companions, individual differences

The goal of this Research Topic was to explore the myriad ways that researchers conceptualize and study the phenomenon of “talking to oneself” and associated experiences of intrapersonal communication. It is clear that people show wide variations in what kinds of intrapersonal communication they experience, how frequently they engage in it, and what functions it serves. In this Research Topic, the contributors explore a range of explanations for how and why people differ in their inner speech, self-talk, or internal dialogue. Our nine contributors examine the phenomenology of intrapersonal communication, its development in childhood, personality and individual differences in the phenomenon, and its occurrence and use in sport contexts.

Variations in intrapersonal communication have been studied using multiple methods, including questionnaires, open-ended self-reports, thinking aloud protocols, imaging techniques, and descriptive experience sampling. Researchers have also started to examine ways that inner speech can be manipulated and the effects of those manipulations on thoughts, emotions, and behavior.

Interest in inner speech (covert self-communication) and private speech (self-communication that occurs aloud) has a long history. However, only recently have researchers begun in earnest to explore the wide range of features of intrapersonal communication. For example, research on various aspects of the neuroanatomy of inner speech and the development of inner speech is very active. Recent work also examines individual and personality differences in the nature, content, and frequency of intrapersonal communication. There is also substantial interest in applied work with self-talk in the domain of sport and athletic performance. This Research Topic highlights work in these areas.

## The Neurological Underpinnings and Development of Intrapersonal Communication

What is inner speech and how does it occur? With the ConDialInt Model, Grandchamp et al. present a neurocognitive predictive control framework of the condensation, dialogical, and intentionality dimensions of inner speech. They illustrate how the form and syntax of inner speech can be condensed or abbreviated, how inner speech can include monologic and dialogic forms involving the self and others, and how it can be produced both spontaneously and willfully. Through an fMRI protocol, they provide neuroanatomical evidence for the intentionality and dialogicality dimensions and how they work together to produce intrapersonal communication.

Geva and Fernyhough highlight the neuro-development of children's inner speech. They show how the dorsal language stream (i.e., the connection between the brain's auditory-phonological and motor systems crucial for speech production) supports the development and phenomenon of inner speech. Their review of pediatric and adult studies of the dorsal language stream supports the idea that there are parallels between the neuro-anatomical and psychological development of inner speech. This overlap suggests that the maturation of the dorsal language pathway is closely linked to the development of inner speech in childhood.

Fernyhough et al. report two studies of the relations among imaginary companions, inner speech, and auditory verbal hallucinations. Noting that imaginary companions in childhood are associated with a variety of positive development outcomes, they compare those with and without such experiences with “hearing voices” and other aspects of inner experience in adulthood. The results showed that, compared to those without a history of imaginary companions, people with such a history reported more frequent auditory verbal hallucinations and higher scores on social-related inner speech. The authors propose that imaginary companions represent a hallucination-like experience that is closely linked to the development of inner speech.

## Personality and Individual Differences in Intrapersonal Communication

Several contributors examined personality and individual differences in intrapersonal communication. For example. Brinthaupt reviews research on individual differences in self-talk frequency according to social isolation and cognitive disruption hypotheses. Individuals who show high levels of experiences of social isolation are expected to show higher levels of self-talk and those with experiences of cognitive disruption (e.g., anomalous, upsetting, or disturbing self-related experiences) should also show increased levels of self-talk frequency. Research provides moderate support for the social isolation and strong support for the cognitive disruption hypotheses.

The relations between inner dialogue types and self-talk functions is the focus of Oleś et al.. They define inner dialogues as intrapersonal communication characterized by different voices and mutual expressions representing self and a wide variety of others and self-talk as self-referent or self-directed speech. Comparing two multidimensional measures of inner dialogue and self-talk among a sample of Polish and US participants, their results show a significant degree of common variance between these two modes of intrapersonal communication. They suggest that inner dialogues appear to serve contemplative or reflective functions of intrapersonal communication, whereas self-talk may serve dynamic, active processing functions.

Łysiak examines the relations between inner dialogues, self-talk, and pathological personality traits, using the DSM-5's new hybrid personality disorder system. She finds that people who report more ruminative and confronting inner dialogues also report higher levels of unusual beliefs, psychoticism, and negative affectivity (e.g., anxiety, separation insecurity). However, specific self-talk facets were unrelated to DSM-5 pathological personality traits. Łysiak suggests that inner dialogues and self-talk are complementary, relating to different aspects of intrapersonal communication.

Finally, Heavey et al. provide a more expansive view of individual differences in intrapersonal communication with the development and validation of their Nevada Inner Experience Questionnaire. The authors show how intrapersonal communication in the form of inner speech is one of several kinds of inner experience, including inner seeing, thinking without symbols, and feelings or emotional experiences. They discuss possible reasons for the relative frequency of these experiences and ways that researchers can increase participants' understanding and awareness of these experiences.

## Intrapersonal Communication in Sport Contexts

Two contributions focused specifically on the role of self-talk in the sport domain. Van Raalte et al. explore how Dialogical Self Theory and the method of Descriptive Experience Sampling (DES) can be used to enhance our understanding of inner experience and self-talk in many sports. They argue that focusing on the dialogical aspects of athlete experiences (such as I-positions and interlocutors, power dynamics, and confrontational vs. integrative inner dialogues) open new avenues for theory and research in sport psychology. DES can provide the tools to assess these theoretical ideas and insights into the phenomenon of athlete self-talk.

Latinjak et al. describe an innovative reflexive self-talk online intervention that targets goal-directed self-talk. During the 4-week program, the researchers encouraged participants to describe challenging scenarios in training or competition, examine how they use self-talk in those situations, determine its effectiveness, and explore alternative kinds of self-talk that they could use in the future. Results showed enhanced awareness of self-talk use and content refinements that appeared to benefit the emotions, motivation, and confidence of the participants. The authors discuss several implications for sport psychologists and other applied practitioners.

In summary, intrapersonal communication is a complex phenomenon, covering concepts such as inner and private speech, self-talk, inner dialogue, and imaginary companions. This topic is an attempt to exemplify the variety of approaches to studying this multi-faceted phenomenon. At the same time, it represents a first step toward a needed synthesis of knowledge about intrapersonal communication. Several unexplored questions remain to be explored, such as whether non-human animals engage in forms of intrapersonal communication and what are the similarities and differences between adaptive and dysfunctional intrapersonal communication. We hope that this selection of articles provides a useful jumping-off point for future intrapersonal communication theorists, researchers, and practitioners.

## Author Contributions

All authors listed have made a substantial, direct and intellectual contribution to the work, and approved it for publication.

## Conflict of Interest

The authors declare that the research was conducted in the absence of any commercial or financial relationships that could be construed as a potential conflict of interest.

